# The effect of acute ingestion of a protein beverage consumed late in the evening on metabolism, appetite, mood state, and blood lipid in overweight and obese adults

**DOI:** 10.1186/1550-2783-9-S1-P16

**Published:** 2012-11-19

**Authors:** Amber W Kinsey, Wyatt R Eddy, Charles J Blay, Takudzwa A Madzima, Lynn B Panton, Jeong-Su Kim, Michael J Ormsbee

**Affiliations:** 1 Department of Nutrition, Food, and Exercise Sciences, The Florida State University, Tallahassee, FL 32306, USA

## Background

Common perception for nocturnal eating has deemed food off-limits during this time due to the potential health implications associated with increased food intake and lack of physical activity during sleep. However, given that macronutrients elicit different effects on metabolism, appetite and cardiometabolic health, it is possible that protein may be optimal for consumption in the evening before sleep. Therefore, the purpose of this study was to investigate the acute impact of protein ingestion consumed in the late evening before sleep on fat metabolism, appetite, mood state, and blood lipids in overweight and obese adults.

## Methods

Forty sedentary overweight or obese (age, 18-45 years), but otherwise healthy, men (n= 8) and women (n= 32) participated in this a placebo-controlled, double blind study. Participants came to the lab fasted (0600-0900) for baseline measurements of appetite ratings (hunger, satiety, desire to eat), mood state, resting metabolic rate (RMR), and blood lipids and glucose. Participants were matched for body fat percent and randomized to one of three groups: carbohydrate placebo (PLA, n= 12; 150 kcals), whey protein (WP, n=14; 150 kcals), or casein protein (CP, n=14; 140 kcals). Participants consumed their respective supplements as the last food or caloric beverage at least 2 hours after dinner but no more than 30 minutes prior to nocturnal sleep. The following morning all participants returned to the laboratory for acute testing to repeat all measurements. Statistical analysis was conducted using 3x2 repeated measures and a Tukey test was used for post hoc comparisons. Significance was set at p<0.05 and all values are reported as means + standard error.

## Results

There were no differences in the dependent variables between groups at baseline as indicated by a one way ANOVA. A repeated measures ANOVA revealed a group by time interaction for higher respiratory quotient (RQ) at baseline in the PLA group compared to the protein groups (p=0.04). Group effects were observed for hunger, RMR, RQ, and glucose. Main effects for time were present for satiety (baseline, 29 ± 2 vs. acute, 37 ± 2) and desire to eat (baseline, 55 ± 2 vs. acute, 47 ± 2). Self-perceived mood indicating more vigor and less confusion in the PLA group compared to the protein groups was reported. All groups had less anger (PLA, baseline, 7.4 ± 1.6 vs. acute, 5.3 ± 1.6; WP, baseline, 9.7 ± 1.5 vs. acute, 6.6 ± 1.5; CP, baseline, 9.6 ± 1.5 vs. acute, 7.6 ± 1.5) and fatigue (PLA, baseline, 8.6 ± 1.1 vs. acute, 7.8 ± 1.1; WP, baseline, 8.8 ± 1.0 vs. acute, 7.9 ± 1.0; CP, baseline, 11.1 ± 1.0 vs. acute, 8.1 ± 1.0) although not statistically significant (p=0.06 for both variables). No differences in blood lipids were present.

## Conclusions

Acute ingestion of a protein beverage consumed in the late evening before sleep does not influence fat metabolism, appetite, mood state, or blood lipids and glucose in overweight and obese adults. Extending the duration of supplementation and including an exercise regimen may provide alternative results and warrants investigation. This study was supported by a grant from FSU’s Council on Research and Creativity.

